# Supporting autistic adults with complex communication needs in making their voices heard: examining an adaptation of the *Autism Voices* framework

**DOI:** 10.3389/fnhum.2025.1638595

**Published:** 2025-11-14

**Authors:** Myriam L. H. Beauchamp, Julie Scorah, Mayada Elsabbagh

**Affiliations:** 1 School of Rehabilitation Sciences – Speech-Language Pathology Programme, Faculty of Health Sciences, University of Ottawa, Ottawa, ON, Canada; 2 Montreal Neurological Institute, Department of Neurology and Neurosurgery, Faculty of Medicine and Health Sciences, McGill University, Montréal, QC, Canada

**Keywords:** autism, complex communication needs, *Autism Voices*, adaptation, inclusion

## Abstract

**Introduction:**

Autistic adults with speech, language and/or cognitive challenges are often excluded from research, particularly from studies examining first-person perspectives, as these generally require that participants have strong speech, language, and cognitive skills. The current pilot study extends previous work and examines whether the *Autism Voices* framework can be adapted for use with a pre-existing interview the *Camberwell Assessment of Need for Adults with Developmental and Intellectual Disabilities*-*Research* version (CANDID-R).

**Methods:**

Eleven young autistic adults with complex communication needs completed the CANDID-R interview using visual supports. These visual supports were provided to assist participants’ comprehension of interview questions and to support them in answering the interview questions. Participants’ caregivers also completed the interview and their answers to specific validation questions were compared to those of their adult children. Additionally, behavioral observations were also completed.

**Results:**

The findings from this pilot study indicate that our adaptation of the *Autism Voices* framework was, at least partially successful in supporting participants in answering the interview questions. Additionally, behavioral observations indicate that the visual supports helped participants remain engaged throughout the interview. However, results also indicate that further adaptations, which we discuss, will be required.

**Conclusion:**

Autistic people with complex communication needs must be included in research about the lived experiences of autistic people. Building on previous work, we show that, with dedication and imagination, equitable and inclusive research is possible.

## 1 Introduction

Autism is a neurodevelopmental condition diagnosed in 1/100 children globally (Zeidan et al., 2022). It is a highly heterogeneous condition, with some individuals presenting strong cognitive and language skills, while others present with co-occurring cognitive disabilities, language disorders and/or speech disorders (American Psychiatric Association, 2013). Historically, research in the field of autism has concentrated almost exclusively on the development of autistic children, with little research focus on autistic adults (Kirby and McDonald, 2021). However, recent research has aimed to better understand the lived experiences, aspirations, wants and needs of these adults, from their first-person perspectives (Nicholas et al., 2019; Tesfaye et al., 2019; Courchesne et al., 2022; Tesfaye et al., 2023).

While this new focus is welcomed and necessary, most of the studies including the first-person perspectives of autistic adults have included individuals without co-existing intellectual disabilities, speech disorders, or language disorders (Nicholas et al., 2019). This fact is concerning since 37.9% of autistic individuals meet the criteria for an intellectual disability (Maenner, 2023), and individuals with important speech disorders (i.e., “minimally verbal”) represent about one third to almost half of the autistic population (depending on the definition of “minimally verbal”; Rose et al., 2016). Because of their exclusion from research, this subgroup has been referred to as the “neglected end of the spectrum” (Tager-Flusberg and Kasari, 2013). This practice is likely explained by the fact that most first-person accounts are gained through verbally conducted interviews, making it challenging for autistic people with complex communication needs (CCN; i.e., people for whom verbal speech is challenging to produce and/or understand; The State of Queensland, 2018; Johnston et al., 2020). Adding to this difficulty is the fact that less than 5% of high schoolers who do not communicate through verbal speech can write simple phrases (Erickson and Geist, 2016), meaning that written expression is not an option for most of these individuals with CCNs. Thus, because of their challenges producing and/or understanding spoken information, and limitations with reading, obtaining the first-person perspectives of people with CCNs is challenging. The exclusion of these individuals from research studies may also stem from an erroneous belief that speech difficulties are always linked to language difficulties (Gernsbacher, 2004) and that these individuals must have too great a language (and/or) intellectual disability to understand interview questions and to give an opinion about their lived experiences.

As a consequence of our neglect of some members of the autism community, little is known about the needs of young adults on the autism spectrum with CCNs from their perspective (Nicholas et al., 2019). While it is likely that individuals on the autism spectrum with CCNs share similar needs to those of autistic individuals without CCNs, it is also possible that their added communication challenges lead to different needs. Moreover, it is unclear the extent to which the perspectives of autistic individuals with CCNs align with those of their caregivers, who traditionally respond on their behalf (Sosnowy et al., 2018; Cheak-Zamora et al., 2017). Finally, it is unclear what is the best way to obtain their first-person perspective.

As Tesfaye et al. (2019) discuss in their review, there exist different ways of eliciting first-person perspectives in autistic people with CCNs. For the most part, studies have used picture-based systems (Nicholas et al., 2019; Tesfaye et al., 2019; Courchesne et al., 2022; Tesfaye et al., 2023) that generally provide picture-based responses options that participants can use to answer interview questions. In addition, some studies also include visual support for the question itself, thus supporting participants who have receptive language challenges to understand the question. For example, in their study Donohue et al. (2014) used the picture-based system Talking Mats (see Murphy and Boa, 2012a,b for description) to ensure that children with intellectual disabilities and verbal speech challenges could answer an in-house questionnaire. Talking Mats can be a useful tool to use in a discrete trial model (such as a questionnaire-based interview). It provides pictograms to support participants’ responses, but also to support their understanding of the question itself. However, questions appear to be depicted in a single pictogram per “mat”, making it more challenging to depict complex questions.

In their study, entitled *Autism Voices*, Tesfaye et al. (2023; also see Courchesne et al., 2022) used a semi-structured in-house interview to elicit the first-person perspectives of autistic youth with and without CCNs regarding their wants, needs, and hopes for the future. To support participants with CCNs understand the interview questions, a set of picture supports was developed for each interview question. Given that some questions included more complex concepts, rather than depicting the question in one picture, several pictograms could be used to depict a single question. A separate set of pictograms was developed to permit those with CCNs provide a response. Thus, participants were able to share their first-person perspective on topics like the future, their need for autonomy, the importantce of their autistic identity and social connection, school, and their mental health.

*Autism Voices* provides a helpful framework that researchers can use to collect first-person perspectives from individuals with CCNs. However, this framework was initially developed for use with a semi-structured interview developed specifically for that study that could be delivered in a very flexible way. It is unclear whether this framework can also be used with more structured questionnaire-based interviews since these validated or norm-based tools permit little leeway in how they are administered or how the questions are asked (i.e., using more adapted language). These realities may make depicting the questions and administering the interview more challenging than when done with an in-house semi-structured interview, the latter being more flexible. Consequently, the current study extends previous work and examines whether visual support strategies based on the *Autism Voices* framework could be adapted for use with a pre-existing research validated standardized interview. We were also interested in examining whether certain participant characteristics were linked to their ability to respond to the interview questions. Finally, this paper provides an in-depth outline of the different steps of the adaptation, the challenges that were encountered and potential solutions to these challenges.

## 2 Materials and methods

This study was part of the Pathways in ASD Project (henceforth the *Pathways project*),^1^ a pan-Canadian longitudinal research project that examines the development of individuals on the autism spectrum from childhood through to early adulthood. Participants received a diagnosis of an autism spectrum disorder when they entered the study (when they were between 24 and 48 months), based on the Diagnostic and Statistical Manual of Mental Disorders, Fourth Edition, Text Revision (DSM-IV) (American Psychiatric Association, 2013), the Autism Diagnostic Interview-Revised (ADI-R) (Nicholas et al., 1994) and the Autism Diagnostic Observation Schedule-Second Edition (ADOS-2) (Lord et al., 2012). The ADI-R and the ADOS were both administered by examiners who were research-reliable. Participants’ diagnosis was also confirmed through the ADOS at several points throughout the Pathways project. To be included in the Pathways project participants had to have a diagnosis of autism spectrum disorder, could not be diagnosed with another genetic or a neurological condition that would prevent them from participating in testing sessions, and their parents were required to speak either French or English (and children had to be exposed to either language).

For the current study, we focused on 11 young adults ranging in age from 20 to 23 years at the time of their assessment (9 male and 2 female). To participate in the current study, autistic participants were required to have a CCN. Functionally, this was defined as (1) participating in an ADOS Module 1 (individuals at pre-verbal or single word level) or 2 (simple phrase speech) at Time point 11 (the previous time point when the ADOS was administered) when participants were 16–18 years-old, and (2) not being able to legally provide independent consent to participate (consent was required from their caregiver, although participants did provide assent). We considered that adults who met these criteria would have challenges producing verbal speech and understanding verbally produced language, and would thus benefit from visual supports to complete the CANDID-R interview. Table 1 contains demographic information, as well as information regarding participants’ cognitive abilities, ADOS scores, communication skills and social skills.

**Table 1:** Demographic information for each participant.

Participants	Age (year; month) at time of current testing	ADOS-2	VABS-2 communication domain	VABS-2 socialization domain	Age (year; month) at time of cognitive testing	WASI PRI	Leiter-3
1	23;0	7	40	75	14;11	75	
2	22;1	7	26	37	14;6		32
3	22;0	7	57	49	14;7	61	
4	21;10	7	40	40	14;5	69	
5	22;0	8	72	82	14;6	62	
6	21;1	6	52	63	13;10	59	
7	21;2	10	43	45	14;5	87	
8	21;5	6	37	49	14;0		51
9	20;0	8	52	56	13;7	90	
10	21;2	6	42	52	14;4		41
11	20;3	6	43	46	13;9		39

Standard scores were used for the VABS-2, the WASI and the Leiter-3. Comparison scores were used for the ADOS-2.

### 2.1 Assessment measures

Autism traits were measured by the Autism Diagnostic Observation Schedule-Second Edition (ADOS-2) (Lord et al., 2012). The ADOS-2 is a semi-structured assessment involving a variety of interactive tasks designed to evaluate social communication, and restricted and repetitive behaviors to inform an autism diagnosis. It yields two main scores: Social Affect and Restrictive and Repetitive Behavior as well as an overall score, with higher scores indicating more autistic traits. Higher scores represent more salient characteristics. The ADOS-2 has 5 modules; a Toddler module (for ages 12–30 months), Module 1 (for individuals who are pre-verbal or have single words), Module 2 (for individuals with simple phrase speech), Module 3 (for children or adolescents with fluent speech), and Module 4 (for adolescents 16 years or older and adults). For comparability of scores across Pathways project sites and time points, the ADOS-2 modules 1 and 2 were administered instead of the research version of the adapted ADOS (A-ADOS) modules 1 and 2, which is a more appropriate test for adults. However, some of the play materials from the adapted ADOS were used to make the administration more age appropriate (for example, the Break toys from the A-ADOS were available during Free Play, the bubble gun was used in place of the bubble blower in Bubble Play, and the helicopter was used in place of foam rockets during the Anticipation of a Routine with Objects task). Overall language and social skills were measured through the Vineland Adaptive Behavior Scales (VABS-2) (Sparrow et al., 2005), Survey Interview Communication and Socialization Domains, respectively. The VABS-2 is a caregiver interview that examines a person’s adaptive skills across several domains. Participants were also administered either the Wechsler Abbreviated Scale of Intelligence-Second Edition (WASI-II) (Wechsler, 2011) or the Leiter International Performance Scale-Third Edition (Leiter-3) (Roid et al., 2013) to measure cognitive ability. For all participants, the WASI-II was attempted first and if participants were able to respond sufficiently to establish a basal score (2 consecutive correct responses) on the Block Design subtest, the WASI-II administration proceeded, and this score was retained as the measure of cognitive ability. Participants who were unable to achieve two correct responses on the WASI-II Block Design task were administered the Leiter-3 as an alternative measure of cognitive ability and the overall score on that test was used as the measure of cognitive ability. In the current study, the *Perceptual Reasoning Index* (PRI) from the WASI-II or the Leiter-Third Edition scores were used as non-verbal IQ (NVIQ) scores. We used NVIQ scores from a previous timepoint (when participants were between 13 and 14;11 years-old), as it is the last time point when NVIQ data was available for all 11 participants. Table 1 contains information related to participants’ age and scores on the different measures.

### 2.2 The interview

As part of the broader Pathways project, we were interested in better understanding whether the needs of young adults on the autism spectrum were being met. To that end, the *Camberwell Assessment of Need for Adults with Developmental and Intellectual Disabilities*-*Research* version (CANDID-R) (Xenitidis et al., 2021) was selected. The CANDID-R is a validated and reliable questionnaire that assesses needs in people with intellectual disabilities and neurodevelopmental conditions such as autism (Xenitidis et al., 2021). It was adapted from the *Camberwell Assessment of Needs* (Phelan et al., 1995) which was developed to assess the needs of individuals with severe mental health conditions. The CANDID is a semi-structured interview; and participants’ responses are categorized according to a discrete set of choices (such as *no need, need met, need unmet, unsure*), based on the seriousness of the problem relative to the help provided. Because of the discrete choice aspect of the CANDID-R, it was appropriate for adaptation using the *Autism Voices* framework. Additionally, the CANDID-R can be completed with both the target participant and with their caregiver separately.

### 2.3 Adaptation of the CANDID-R

Two visual-support documents were created: the first to support participants’ understanding of the CANDID-R questions (question document). The second document provided visual supports for the response options to each question (response document). The development of these documents was an iterative process consisting of several rounds of revisions for both the question and response documents which were completed by a team comprised of a research assistant, a Speech Language Pathologist (MB), and a Neuropsychologist (JS). Visuals were created using Boardmaker 7 Editor, a software application that enables the creation of visual supports. All visuals were printed and presented in full color.

To depict the CANDID-R questions and response options, we initially attempted to search for images to represent key concepts in the question to convey meaning. For example, the question “do you make your own meals?” was represented using an image to convey the idea “you,” another to illustrate the concept “make” and a third to represent “meals.”

**Figure 1:**
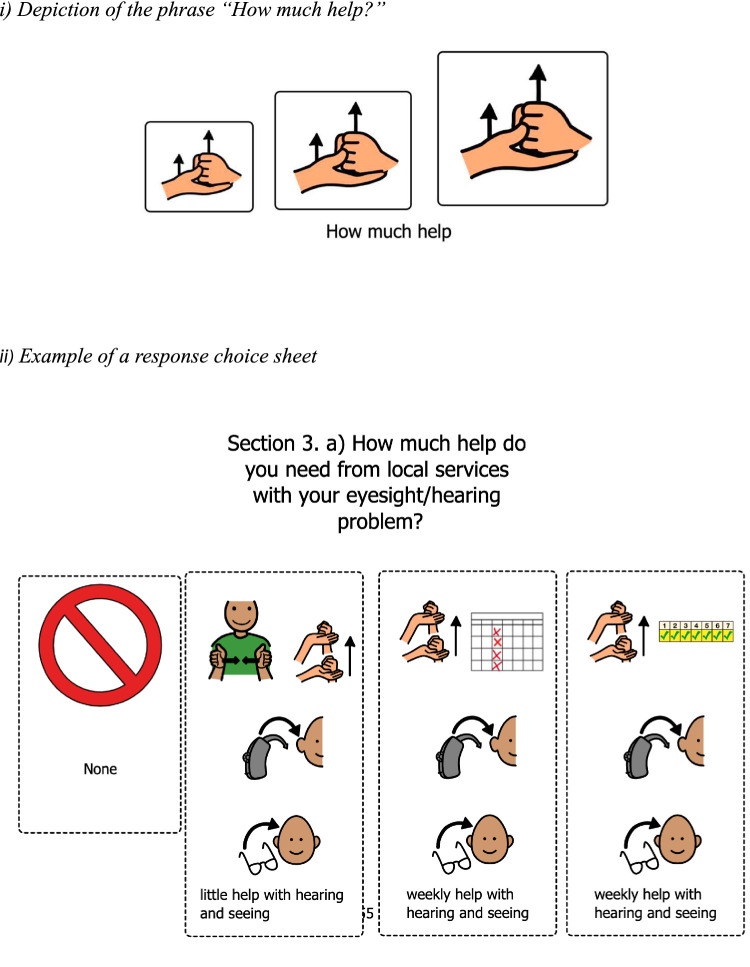
Examples of visual supports.

Abstract concepts were more challenging to depict using our initial strategy. Thus, we aimed to depict the meaning of the different phrases within the question. For example, the phrase “how much help” was depicted using 3 pictograms: a small, medium, and large image of the American Sign Language (ASL) sign for “help” to represent “a small amount of help,” “a medium amount of help,” and “a large amount of help.” Although the concept “how much help” is fairly straightforward, other concepts were much more challenging to depict. For example, the term “exploitation/abuse” was initially depicted with an image of one individual hitting another, which we felt was not sufficient to represent this concept. Thus, we broke down the term into concepts, identified the underlying meaning of these concepts, and identified words linked to the ideas linked to this meaning. These words were then used to search for images that best represented the meaning the concept. In doing so, we aimed to depict the meaning of a question, rather than find an image to accompany specific words within the question. In addition, while we did use some sign language symbols, particularly those that are commonly taught (e.g., “help”) we tried to avoid their use as they are not universally taught and since the symbol for a given concept may vary from one sign language to another (e.g., Langue des signes du Québec vs. American Sign Language). Finally, we were careful to ensure that pictograms depicting a term/idea could only be used for that specific term/idea. Examples of the visual supports for the questions and response can be found in Figure 1.

### 2.4 Pre-pilot testing session

A pre-pilot testing session was completed with an autistic young adult who met our participation criteria. This session permitted the examiner (JS) to assess the feasibility of administering the CANDID-R with our visual supports in the way that we had initially envisioned doing so, and to make adjustments where necessary.

Initially, we sought for the administration of the CANDID-R to be as flexible as possible, mirroring the strategy used in the original *Autism Voices* study (Courchesne et al., 2022; Tesfaye et al., 2023). To that end, we had planned to present each set of pictograms (linked to a question or response choices) as a separate picture card. This strategy would have made it easier to break down ideas and explain the question, and would have given us the flexibility to move the response cards around (thus counter-balancing the location of the response cards). However, as we discovered during the pre-pilot testing session, given the large number of questions in the CANDID-R, and therefore the large number of pictograms, this strategy was not feasible. As a result, it was decided that each question and set of response choices (along with their respective accompanying pictograms) would be printed on its own sheet of paper (one for the question and one for the response choice). These sheets were placed in a large binder, such that question sheets would be shown on the top page of the open binder while at the same time the response choices would be shown along the bottom page of the open binder, with all visuals oriented toward the participant. This pre-pilot test session also allowed us to test our behavioral coding scheme, which we also realized needed to be adjusted. Thus, we refined the coding scheme to determine simply whether the participant was attending to each question.

Since we wanted this administration to closely mirror how the administration of the CANDID-R was completed with other autistic participants without CCNs (who were part of the broader Pathways study), there were no warm-up activities or additional layers of support. We also opted for this approach since the goal of this study was to examine if we could use the *Autism Voices* framework while remaining faithful to the CANDID-R’s administration protocol. Finally, while the CANDID-R does permit some rephrasing of the questions, we were mindful to always first present the question as it was written and to limit significantly rephrasing the questions for two reasons (1) other similar types of interviews do not permit rephrasing, and (2) to avoid losing the intended meaning of the original question.

### 2.5 Testing sessions

Participants were administered the CANDID-R and the ADOS-2 by a neuropsychologist (JS who is research reliable on the ADOS-2) during a single session either at home or in an evaluation room at the Montreal Neurological Institute (MNI). Home visits were offered when caregivers reported having difficulty coming to the MNI due to transportation issues (not having a car, insufficient time to commute) or that their adult child’s anxiety or behavior in a novel setting would impede valid assessment. Most participants were evaluated alone in the room with the examiner, but in some cases the caregiver was in the room during the interview for the comfort of the participant. Caregivers who were present during the interviews were instructed not to answer for their adult child. The VABS-2 was administered to the caregiver most knowledgeable about their adult child by a trained research assistant via a phone interview, generally preceding the participants’ direct testing session. Caregivers also completed the CANDID-R without visual supports, during a virtual interview session with a neuropsychologist.

For the adapted CANDID-R administration (administration with visual supports), participants were seated facing the binder containing the *question* and *response* sheets. Each sheet contained the written question, the visual supports (pictograms) and wording under each set of pictograms linking them to the question (see examples in Figure 1). First, the examiner read the question while pointing to each related pictogram/set of pictograms. Next the examiner read out the response options, again while pointing to each related pictogram. If the participant appeared distracted while the question was being asked, the examiner could reread the question. The examiner could also slightly rephrase or bring a participant’s attention to certain concepts as required. Participants could answer the question using words, a full or partial point to their response choice pictogram, by tapping their response choice pictogram or using gestures. These were the only answers recorded by the examiner. Participants were permitted to bring with them items that helped with sensory integration, transition items, or any other item that would support their participation and limit anxiety or stress during the testing session. Each adapted-CANDID-R interview session lasted between 30 and 45 min and all sessions were video recorded.

### 2.6 Validation strategies and planned analyses

We validated our adaptation by selecting five questions that required a factual answer (as opposed to a response regarding one’s own perceptions or wishes) and comparing participants’ responses to their caregivers’ responses. This strategy helped us determine whether participants understood the questions and were able to provide accurate answers. Because the CANDID-R examines people’s opinions regarding needs met, the answers to most questions do not permit comparisons of factual information thus limiting the questions that could be used for validation purposes. Additionally, during direct testing sessions, the examiner, who is a licensed neuropsychologist with 20 years of experience in assessing autistic people (JS), recorded the participant’s level of attention (scale of 1–3; 1 = not attending, 2 = somewhat attending, 3 = attends well), which was defined as participants’ looking at the visual supports for the questions and response options, and shifting their gaze to the appropriate set of pictograms as the examiner read the question and response options. The examiner also made note of any response patterns or behaviors that could be informative. Next, the first author (MB), who is a licensed speech-language pathologist with 14 years of experience working with and assessing autistic people, reviewed the video recording of each participant’s testing session and independently noted each participant’s level of attention and described response patterns and behaviors. These observations were not used to infer a participant’s answers to the CANDID-R questions, but rather to help inform whether further changes to the adaptation would be required.

For each participant two mean attention level scores were calculated: one based on the examiner’s scores and one based on the first author’s scores. To do so, the attention scores received for each question were added and the total divided by the number of questions asked (which could differ across participants) to wield a score out of three. Finally, we examined whether there was a correlation between participants’ social and communication skills, (as measured by the VABS-2), autistic traits (as measured by the ADOS-2) and the number of concordant answers between each participant and their caregiver out of the five validation questions. To that end, we completed a Bayesian correlation using JASP (JASP Team, 2025) and used Bayes factor (BF) to examine the likelihood/probability that the number of correctly answered questions was correlated to participants’ social and communication skills, and autism characteristics (Brydges and Gaeta, 2019). We used the BF_10_ model where a BF above 1 supports the hypothesis (a correlation between questions answered and participants’ characteristics), a BF below 1 supports the null hypothesis, and a BF of 1 suggests that both hypotheses are equally plausible (van Doorn et al., 2021).

## 3 Results

To examine whether our adaptations were helpful in providing support to young autistic adults in responding to the adapted- CANDID-R questions, we first examined participants’ answers to the five validation questions compared to those of their caregivers. An analysis of participants’ and caregivers’ responses revealed that they both answered the validation questions in 65.38% of opportunities. When a response was not obtained, in all cases, it was the young adult who did not respond to the question. When including responses and no-response to the validation questions, young adults’ and caregivers’ responses were concordant 46.15% of the time. However, when we only considered responses to the questions that were answered, young adults and caregivers gave concordant responses to the validation questions in 70.59% of opportunities. Of the 11 young adult participants in this study, five consistently provided responses to the validation questions that were concordant with those of their caregiver. Also, an examination of young adults’ responses to the validation questions indicated that not all questions were equally understood. For example, the question regarding whether participants had mobility issues was concordant in 87.5% of cases, suggesting that the young adults mostly understood the question. In contrast, only 50% of the young adults gave a correct response (i.e., concordant with their caregiver’s response) to the question asking whether they had children to care for (see Table 2 for each participant-caregiver pair’s performance on the validation questions).

**Table 2:** Performance on validation questions and levels of attention.

Participants	Number of concordant question pairs out of the total validations questions asked	Number of concordant questions pairs out of total number of questions answered by the participant	Mean level of attention during the session (out of 3) Examiner/first author
1	2/5	2/2	2.95/2.97
2	1/5	1/1	1.96/2.6
3	4/5	4/5	2.84/2.44
4	4/5	4/4	3/2.9
5	2/5	2/4	2.87/2.79
6	3/5	3/5	3/3
7	2/5	2/5	2.84/2.04
8	3/4	3/3	2.92/2.91
9	2/3	2/3	2.95/2.99
10	0/5	0/1	2.8/ 2.58
11	1/5	1/1	1.85/2.11

Two participant-caregiver pairs were not asked all five validation questions.

We also examined participants’ levels of attention during the session. Nine of the 11 participants received an overall attention score in the “somewhat good” (score of 2) to “good” (score of 3) range by both the examiner and the first author, suggesting that most participants attended well during the administration of the adapted-CANDID-R (Table 2). It was noted that the two participants who received attention scores under 2 from the examiner, presented with more frequent repetitive and sensory-related behaviors.

To examine the possible link between participants’ performances and their language and social skills, two sets of Bayesian correlations were completed with JASP; the first using the number of concordant responses out of the total number of validation questions (including questions to which participants did not respond), and the second using the number of concordant responses out of the number of validation questions answered by both the participant and their caregiver. In both cases participants’ standard scores on the Communication and the Socialization domains of the VABS-2, and the ADOS-2 Comparison scores were the independent variables. Since two participants did not receive all five questions, the number correct over the number of questions asked was converted to a percent correct score. As Tables 3, 4 show the BF_10_ indicates that for both sets of analyses the null hypothesis was supported across all variables. Thus, it is unlikely that the dependent and independent variables are correlated.

**Table 3:** Bayesian Pearson correlations between the percent of concordant responses on validation questions (answered and not answered) and language, social skills and ADOS scores.

Variable		VABS communication	VABS socialization	ADOS-2
Percent concordant responses	Pearson’s r	0.245	−0.032	0.073
	BF₁₀	0.468	0.371	0.377

*n* = 11.

**Table 4:** Bayesian Pearson correlations between the percent of concordant responses on validation questions (only questions both caregivers and participants answered) and language, social skills and ADOS scores.

Variable		VABS communication standard	VABS socialization standard	ADOS-2 comparison score
Percent concordant responses	Pearson’s r	−0.361	−0.212	−0.238
	BF₁₀	0.631	0.441	0.462

*n* = 11.

**Figure 2:**
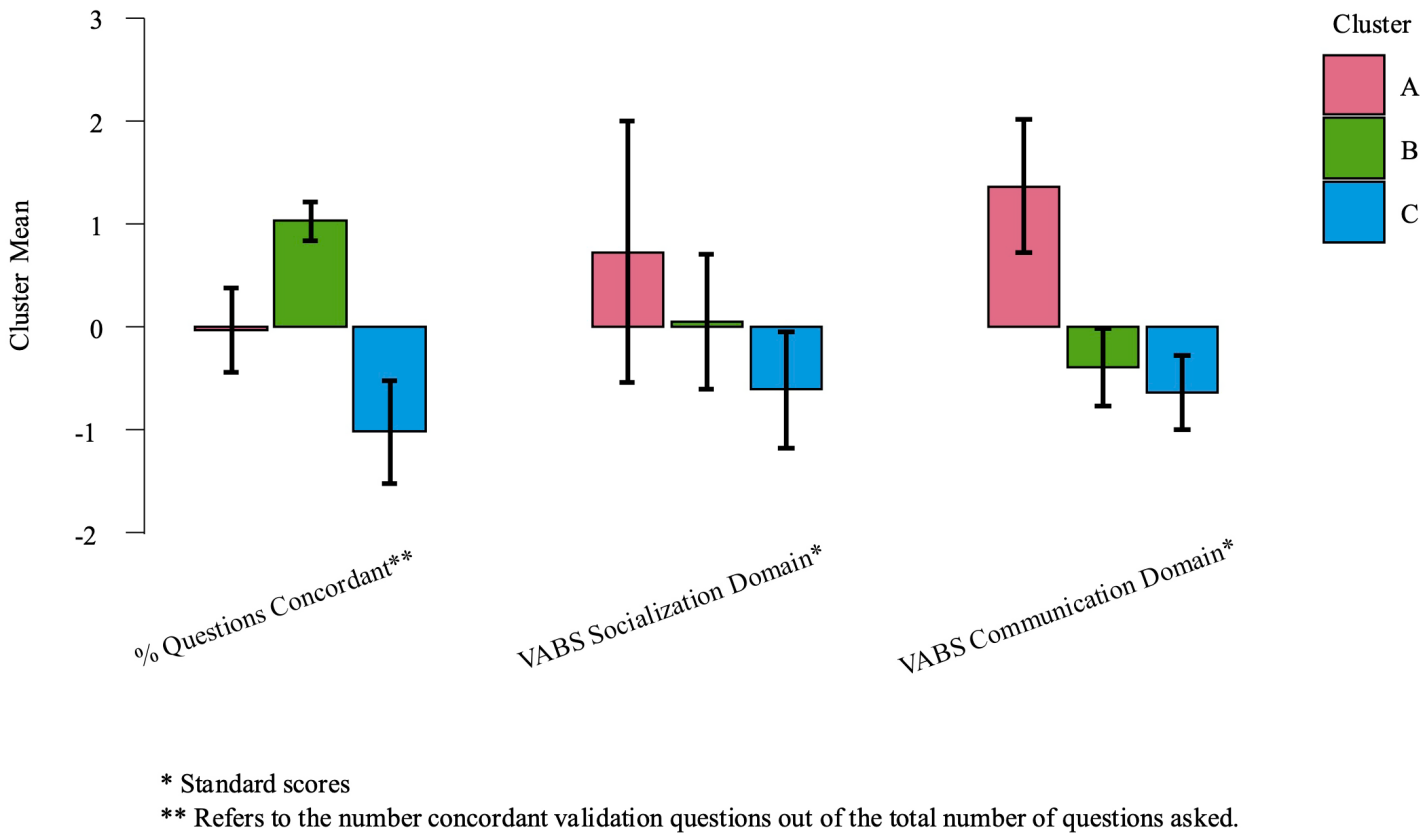
Cluster analysis using the percent of concordant validation question, and VABS socialization and communication domain scores.

Since no one independent variable was likely correlated to the percent of correctly answered validation questions, we completed a post-hoc cluster analysis to examine whether certain groups of characteristics were linked to (i.e., grouped with) participants’ ability to correctly respond to the validation questions. The analysis was completed using JASP K-Means Clustering and a “best fit” approach with the Bayesian Information Criterion (BIC) option, and entering the percent of concordant pairs of validation questions out of the total number asked, as well as the composite scores for the VABS Socialization Domain and the Communication Domain. As the bar plot (Figure 2) indicates, Cluster A includes individuals with average percent concordant performances and high scores on the Socialization and Communication domains, Cluster B includes participants who had high percent concordant performances, average Socialization scores and somewhat low Communication scores and Cluster C included participants with the lowest performances/scores for all 3 variables.

### 3.1 Observations

Because most of the CANDID-R questions require that participants give their opinion regarding their needs, which may not be shared by their caregiver, comparing participants’ responses to those of their caregiver was not possible beyond the five validation questions. However, an examination of participants’ behaviors and response patterns (through direct and indirect [video] observations) provided important insights regarding our main research question. First, we examined participants’ responses to the questions asking (1) if the participant is receiving the right type of help/services and (2) if the participant is happy/satisfied with the help/services that they were receiving. Since these questions were asked in all 25 sections of the CANDID-R, participants’ responses provided insight as to whether the visual supports were adequate for more subtle differences in meaning. Participants’ response patterns revealed that five participants consistently gave the same answer to both of these questions across all of the sections, indicating that they might not understand the difference between these two questions. Additionally, on a few occasions, some participants provided a different answer to each of these questions, but their responses were not logical: they answered “no” to the first question (getting the right type of help) but “yes” to the second question (satisfied with the help that they are getting). Thus, participants’ response patterns suggest the current visual supports may be insufficient to support most participants’ understanding of these subtle differences.

Observations also indicated, as noted above, that participants did not always provide a response to the CANDID-R questions. However, some participants’ behaviors during the interview seem to indicate that even when they did not respond to a question, they may nevertheless have understood the theme of the question. For example, one participant giggled when the examiner asked a question around sexuality, even though they did not provide a response. Other participants made signs and gestures linked to the question. For instance, one participant pretended to bite their arm when asked a question about self-harm and mimed a seizure for a question on that topic. Another participant made the ASL sign for *eating* to a question about having enough food to eat. Such behaviors suggest that the participants understood at least some aspects of the question. Thus, the visual supports appear to promote a certain level of comprehension of the CANDID-R questions. Moreover, for some participants, the act of indicating their choice by pointing to/touching a picture was difficult or unclear. For example, one participant’s behaviors throughout the assessment suggested that they were attending to the examiner’s questions (looked intently at the visual supports and back to the examiner) and perhaps also understood the questions (made facial expressions that could indicate that the were thinking about the question). However, the participant consistently looked to their caregiver and grabbed the caregiver’s hand in order to get their caregiver to respond to the question. The participant’s intent was confirmed by the caregiver, who commented that their adult child wanted them (the caregiver) to answer the question. Minimally, this behavior suggests that this young adult understood that the examiner was asking a question and that a response was required.

Observations also indicated that challenges in inhibiting repetitive behaviors may have interfered with some participants’ ability to respond to the CANDID-R questions. For example, one participant consistently pointed to all of the pictograms on the response sheet and then often double-tapped the final picture. Initially this double-tap was interpreted as a response. However, it became clear that this assumption was incorrect since they (i) always double-tapped on the last picture (regardless of the picture) and (ii) at times, their “response” contradicted the answer that they had given to a previous question. Another participant frequently banged on the table and on the response sheet. It was unclear whether the latter was an attempt by the participant to provide an answer since the tapping was repetitive and did not appear to be on a specific picture. Other participants focused intently on specific pictograms. One participant was drawn to the pictogram of someone vacuuming and would say the word “vacuum” every time they saw this visual. Another participant focused on the pictogram for “help”, consistently pointing to that visual and saying “help” when they saw it. For these participants’ their preference for a given pictogram seemed to frequently interfere with their attention to the question. Similarly, some participants tended to echo what the examiner said. In these cases, it was difficult to reliably parse out the participant’s intentional response from a repetitive behavior. Finally, four participants seemed to read the questions and the words under the pictograms. While for some participants this ability might have been helpful, for others the wording seemed distracting. Moreover, it was unclear whether these participants were truly reading (i.e., decoding plus comprehending the message) or simply decoding what was written on the page. Nevertheless, in all cases where a response was provided, the participant’s responses were recorded. The examiner abstained from interpreting the participant’s intent when recording their answers.

Overall, observations revealed that participants respond positively to the CANDID-R adaptations. One participant who had some speech abilities and the caregiver of another young adult commented positively on the visual supports, both indicating that these supports were helpful. Although one participant did report being bored during the interview, but nevertheless agreed to complete it. The visual supports also seemed to increase participants’ ability to actively attend to the examiner’s questions. For example, two caregivers expressed some surprise that their adult child remained seated and attentive throughout the interview, since it is usually challenging for them to do so during non-screen activities, attributing this sustained attention to the visual supports. These caregivers also expressed an interest in starting to use similar visuals at home. The examiner also noted that, participants were generally more attentive during the administration of the adapted-CANDID-R than they were during other portions of the testing session. For example, prior to the administration of the interview, some participants displayed high amounts of repetitive and at times destructive behaviors upon entering the testing area. These included repeated loud yelling, forceful slapping of furniture and of others, rummaging in drawers and cabinets, knocking materials off tables and walls, tearing up or breaking materials, etc. (which participants’ caregivers reported were common in novel settings). However, once these participants were seated and presented with the visual supports, they appeared interested, and their activity level and repetitive behaviors decreased such that they were able to complete the testing session. In fact, all 11 participants remained seated and as demonstrated by their attention scores, were reasonably attentive throughout most, if not all of the adapted-CANDID-R interview, with none of the participants failing to complete the entire interview.

## 4 Discussion

The goal of this study was to examine whether we could adapt the *Autism Voices* framework for use with the CANDID-R with young autistic adults with CCNs. Overall, results suggest that our adaptation was at least partially effective in supporting these participants’s ability to express their needs through a semi-structured interview, as evidenced by their responses to the validation questions, as a group, they were more likely to answer factual questions correctly (i.e., provided a response that aligned with their caregiver’s response). This finding is encouraging as it indicates that autistic people with CCNs can participate in a meaningful way to interviews such as the CANDID-R when they receive appropriate visual supports. Observations revealed that throughout the interview session, which was between 30 and 45 minutes in length, the majority of participants attended somewhat to very well. Furthermore, several participants displayed more focused and attentive behavior during the interview than during other portions of their testing session. Additionally, a participant and a caregiver spontaneously indicated liking the pictograms and feeling that they were helpful. Two caregivers also noted that the visual supports helped their child attend and that they would like to use similar pictograms at home to support their child’s communication. Together, these behavioral patterns and feedback indicate that the visual supports are engaging and helpful and might also suggest a certain level of acceptability of our adaptation by participants.

Nevertheless, our findings also indicate that, in many cases, participants presented difficulties providing a response to the CANDID-R questions, even with the visual supports and revealed several challenges in adapting and administering the CANDID-R using an adaptation of the *Autism Voices* framework. In the following paragraphs we discuss the possible reasons why participants may have experienced challenges in providing responses, the challenges that we encountered during the administration phase, and potential solutions to mitigate these issues where appropriate.

One possible explanation for participants’ difficulties with responding to the CANDID-R question may be that our visual supports were insufficient to promoting their understanding of the interview items. Indeed, direct and indirect observations revealed that throughout the adapted-CANDID-R interview, some abstract concepts such as “having children” were consistently difficult for participants to comprehend, even with visual supports. Additionally, pairs of questions that had closely related but distinct meanings such as *what I am receiving* and *what I would like to receive* were also challenging for participants. These findings indicate, at least in part, that further refinement of our visual supports is necessary to better facilitate participants’ understanding of these concepts. To that end, we plan to develop an additional set of visual supports that more fully explain these concepts which could be presented to participants prior to asking questions containing the target concept.

While improving our visual supports is an important step, observations revealed other challenges that require mitigation. First, some, participants’ performances seemed to indicate a potential discrepancy between their understanding of the questions and their ability to provide a response. These participants demonstrated being attentive, and seemed to minimally understand the main theme of the question, or at least that a question was asked and that a response was expected. Nevertheless, it was difficult for them to provide a response. This behavior was noted even when a participant seemed to be looking at a specific response picture. It is possible that some participants simply had difficulty understanding how to provide a response, particularly if pointing does not come naturally to them. However, since these individuals were evaluated several times over the course of the Pathway project and because, for this study, we accepted many different forms of responses (saying, tapping, pointing, gestures) this challenge was not anticipated. Some caregivers reported that their adult child no longer engaged as readily, or participated as actively, in tabletop activities as they had previously. They attributed this decrease in motivation for table-top tasks to no longer being in a school environment. To mitigate this potential challenge moving forward. We plan to add training items to the research protocol, thus adding a teaching component to the testing session.

Second, for some participants, repetitive behaviors such as touching all the pictograms, seemed to hinder their ability to answer the questions. Direct and indirect observation revealed that some of these behaviors were, at least initially, an imitation of the examiner who pointed to each response choice as she named them to help bring the participant’s attention to the appropriate pictogram(s). To help limit these types of behaviors, we plan to develop a computerized version of our adaptation using touch screen technology. For both the question and response choices, each pictogram (or set of pictograms when several depict one concept) would become more salient as it is spoken to bring the participant’s attention to that pictogram without an examiner needing to point to it. For participants for whom having written text is a distraction rather than additional support, a computerized version of our adaptation would also permit us to remove the text as needed. Finally voice-generated technology could be used to speak the question and response choices. In doing so, we would control the rate of speech from one participant to another.

It is important to recognize that while visual supports are helpful, they have several limitations. Indeed, one’s mental representation of a particular concept is formed (at least in part) from our lived experiences (Barsalou, 2010; Gopnik and Meltzoff, 1997; Ullman and Tenenbaum, 2020). It is therefore possible that people have different mental representations of the same concept because of differences in lived experiences. For example, one person’s representation of an apple may be *a red apple* because it is the type of apple that they have always eaten. But another person’s representation of an apple might be *a green apple* because they had an apple tree in their backyard that grows green apples. While this example is admittedly overly simplistic, it does demonstrate that when visual supports are developed, they represent the developer’s mental representation of a concept, which may be different from another person’s mental representation of a concept. In individuals who have difficulty with cognitive flexibility, as is reported in some autistic individuals (Lage et al., 2024), it is unclear whether, or the extent to which adjusting their mental representation of a concept to another person’s representation of that concept is challenging. Moreover, developing a mental representation of a concept requires, à priori, that one be exposed (either directly or indirectly) to this concept (Barsalou, 2010; Gopnik and Meltzoff, 1997; Ullman and Tenenbaum, 2020). Consequently, young adults who have never or rarely been exposed to concepts like paying bills, managing money or applying for welfare benefits may not have a mental representation for such concepts given their lived experiences. Thus, responding to questions for which they do not have a conceptual representation would be challenging for these individuals, regardless of the presence or quality of the related visual supports. While we may not be able to mitigate this challenge directly, it will be important moving forward to gather information from caregivers as to their adult child’s exposure to these concepts. With this information in hand, we could better judge the reliability of participants’ responses to various interview questions.

Although all the participants in this study had CCNs, they also represent a wide range of non-verbal cognitive, language and speech abilities (with most of our sample scoring in the range of intellectual disability). Because of their challenges in these domains, many of the participants in this study would traditionally have been excluded from participating in research, particularly when the study design requires good language, speech and cognitive skills. Yet, as our results demonstrate, autistic individuals with CCNs can participate in research in a meaningful way and share their lived experiences. However, their inclusion requires a protocol that is pragmatic and flexible while respecting the limitations that come with using a research-validated tool for research purposes. Thus, both the adaptation of the tool and its administration require time and expertise.

### 4.1 Limitations

Given the pilot and exploratory nature of our study, it does have several limitations. First, it only included 11 participants. While this study revealed important information and challenges, we acknowledge that our findings are not generalizable. Next, given the nature of the CANDID-R, few questions could be used to validate our adaptation. In future studies, we will need to consider other ways to validate our adaptation and participants’ answers that do not add additional time requirements on participants and their families. Additionally, it was not possible to examine the influence of participants’ cognitive skills on their performances since not all participants in our sample completed the same test (i.e., some did the WASI, while others did the Leiter). However, a visual examination of the number of correct responses to the validation questions and NVIQ scores seems to indicate that cognitive skills did not account for participants’ abilities to correctly answer the CANDID-R questions. Other participant characteristics could potentially affect our findings, but we believe that it is imprudent to speculate on what these could be and how they could affect our findings given the small number of participants in the current study. Finally, developing a protocol that permits the inclusion of people with CCNs but also respects the limits of research-validated tools (like the CANDID-R) required a significant amount of time and clinical expertise. While the latter is a strength of our study, it is a cautionary note. Together, the first and second authors have over 30 years of clinical experience working with autistic people, including a speech-language pathologist who has experience developing and using visual supports with autistic people who have CCNs. This expertise was crucial in developing and testing our adaptation. Even so, we still faced many unforeseen challenges. In light of this fact, it is critically important that research teams who endeavor to adapt an existing interview using visual supports include team members with the expertise required to do so: ideally, (minimally) a speech-language pathologist with expertise working with autistic people and developing and using augmentative and alternative communication (AAC) methods, and/or other clinicians with similar expertise and experience.

## 5 Conclusion

The results from this study, while not fully satisfactory, are encouraging and demonstrate that the *Autism Voices* framework can be used to support the participation of autistic individuals with CCNs in completing existing interviews like the CANDID-R. Our findings also indicate that autistic people with CCNs can participate in research and share their lived experiences. However, this inclusive approach requires that researchers be creative in developing and refining methods to include the voices of all autistic people.

## Data Availability

The raw data supporting the conclusions of this article will be made available by the authors, without undue reservation.

## Footnotes

^1^ http://www.asdpathways.ca
